# Chemical Diversity of Wild-Growing and Cultivated Common Valerian (*Valeriana officinalis* L. s.l.) Originating from Poland

**DOI:** 10.3390/molecules29010112

**Published:** 2023-12-23

**Authors:** Kavana Raj, Zenon Węglarz, Jarosław L. Przybył, Olga Kosakowska, Anna Pawełczak, Łukasz Gontar, Marta Puchta-Jasińska, Katarzyna Bączek

**Affiliations:** 1 Department of Vegetable and Medicinal Plants, Institute of Horticultural Sciences, Warsaw University of Life Sciences SGGW, 159 Nowoursynowska Street, 02-776 Warsaw, Poland; kavana_raj@sggw.edu.pl (K.R.); zenon_weglarz@sggw.edu.pl (Z.W.); jaroslaw_przybyl@sggw.edu.pl (J.L.P.); olga_kosakowska@sggw.edu.pl (O.K.); anna_pawelczak@sggw.edu.pl (A.P.); 2 Research and Innovation Centre Pro-Akademia, 9/11 Innowacyjna Street, 95-050 Konstantynów Łódzki, Poland; gontar.lukasz@gmail.com; 3 Plant Breeding and Acclimatization Institute, Radzikow, 05-870 Blonie, Poland; m.puchta@ihar.edu.pl

**Keywords:** ‘Lubelski’, underground organs, ploidy, valerenic acids, essential oils

## Abstract

Common valerian is a medicinal plant. The underground organs of this species are used as a mild sedative and sleeping aid. Poland is one of the largest producers of this raw material in Europe, with local cultivar ‘Lubelski’ as a primary cultivated form. Although valerian is the subject of more or less deliberate selection carried out by farmers, it is still genetically unstable. The aim of this study was to determine the diversity of the ‘Lubelski’ cultivar originating from four regions of Poland (forms: L1–L4) in relation to wild-growing populations of the species. The plants were assessed in terms of the mass of underground organs and the content of valerenic acids and essential oils (EOs). The content of valerenic acids was determined using HPLC, whereas the content of EOs was determined using hydrodistillation. The composition of EOs was assessed using GC-MS GC-FID. The ploidy level of the analyzed objects was determined as well. Wild-growing populations (diploids) were characterized by lower masses of underground organs and lower contents of valerenic acid than cultivated forms (tetraploids). However, they produced higher contents of EOs. All the cultivated forms were strongly diversified with respect to the analyzed traits, including the mass of the roots (CV 49–75%), the content of valerenic acids (CV 18–55%), and the content of EOs (CV 28–57%). A total of 44 compounds were identified in the EOs. The dominant compound of both wild-growing populations and the ‘Lubelski’ forms were: α-fenchene, bornyl acetate, and valerenal. Among ‘Lubelski’ forms, the most interesting seems to be the L2 form, which was characterized by a relatively high yield and high content of valerenic acids and EOs. Thus, it appears to be a promising source of objects for further valerian cultivar improvement.

## 1. Introduction

Wild-growing common valerian (*Valeriana officinalis* L. s.l.; Valerianaceae) originates from temperate regions of Europe and Asia. In North America, it has been naturalized. Its populations are found on riverbanks, in wet meadows and low peat bogs, on sunny edges of forests (usually dominated by alder), amid scrub, and on roadsides. Being extremely polymorphous, *V. officinalis* constitutes an aggregate taxon with a complex of subspecies. Up to now, three basic ploidy levels have been identified within the species: diploid, tetraploid, and octoploid. Aneuploidy was detected among some Italian populations as well [1,2,3]. Common valerian develops as a rhizomatous rosette-forming clonal perennial plant with solitary hollow stems reaching 2 m in height, with pale pink to white strongly scented flowers at the top [4].

The herbal raw materials collected from this species are underground organs consisting of a vertical rhizome bearing numerous roots and some stolons, commonly named valerian root. Currently, the raw material from which the medicinal products are produced originates exclusively from cultivation and is subjected to standardization. According to the European Pharmacopoeia [5], the dried, uncut, or fragmented valerian root (*Valerianae radix*) should contain no less than 0.17% sesquiterpenic acids (commonly named valerenic acids) and no less than 4 mL × kg^−1^ DW of essential oil (EO). In Europe, the drug is applied based on both well-established and traditional use [6]. Preparations thereof are available here mainly in the form of dry extracts (extraction solvent: ethanol 40–70% *v/v*). Other forms of valerian preparation are also in use, including comminuted or powdered drug used to prepare an infusion, juice from fresh roots, and diverse liquid extracts and tinctures for oral use. Comminuted herbal raw material or preparations thereof are also used in liquid forms as a bath additive [6]. In turn, in the US and Canada valerian root is approved as a dietary supplement (considered as food), being generally recognized as a traditional herbal sleep aid. With reference to the EMA/HMPC monograph, valerian preparations are indicated for the relief of mild nervous tension and sleep disorders (according to well-established use) and for the relief of mild symptoms of mental stress and as sleep aid (according to traditional use). Moreover, valerian root products show no risk of forming dependence as may often occur with synthetic sedatives. Such important effects of these preparations make them one of the most frequently purchased herbal remedies in Europe and the US [7,8]. However, it is still not possible to demonstrate a clear relationship between the clinically confirmed efficacy of valerian as a sleep-promoting drug and specific compounds present in this raw material. This efficacy probably depends on an interplay between many groups of compounds [9]. These include EO (0.1–2.8%), consisting of a mixture of mono- and sesquiterpenoids. The major monoterpenes here are borneol and its isovaleric and acetyl esters or acetates. As for sesquiterpenes, the major specific ring systems unique to the Valerianaceae are visible in the valerian EO. These include kessane and elemane ring types, of which valeranone is an important compound, as well as valerenal and valerenic acids. Valerian root contains also 0.1–2.0% valepotriates (monoterpenes belonging to the iridoid group), with valtrate and isovaltrate being dominant, and characteristic-free sesquiterpenic acids such as valerenic and hydroxyvalerenic acids, which have so far not been detected in any other species than *V. officinalis*. Thus, their presence is used in drug identification. Moreover, the raw material contains some lignans (about 0.2%), traces of alkaloids, phenolic acids, and fatty acids and their esters [10,11,12,13].

For centuries, valerian root was collected from wild-growing plants. As mentioned above, nowadays, the material for commerce originates exclusively from cultivation, mainly from Poland, Belgium, The Netherlands, Germany, the Czech Republic, Ukraine, Hungary, and the United States. The varieties used for crop production are represented predominantly by improved populations created using simple breeding selection or crossbreeding methods [14]. In Poland, the species was introduced into broader cultivation in the 1950s. Since then, farmers have carried out their own selection based on wild-growing populations (usually diploid forms), mainly in terms of root weights, in order to improve the yield. As a result of this work, a local cultivar was created and named ‘Lubelski’ after the region where it had been developed. The cultivar was identified later as *V. officinalis* subsp. *latifolia* (*tenuifolia*) and classified as a tetraploid characterized by thick roots and a high content of essential oil [15]. ‘Lubelski’ is still being improved by farmers and is widely cultivated in Poland and neighboring countries. However, farmers’ own seed reproduction, combined with the selection of plants intended for this purpose, carried out locally in various regions of Poland, contributes to increasing the range of variability in this population variety and creating its local forms (subpopulations). Thus, such population varieties show high heterogeneity in the yield and content of active constituents in the raw material [14].

In order to undertake further breeding programs aimed at improving yield-creating traits and levelling the content of active compounds in raw materials obtained from such population varieties, first it is necessary to assess the range of diversity of the forms used so far. The aim of the study was to determine the range of the variability both within and between ‘Lubelski’ forms of valerian originating from four remote cultivation regions of the plant, with reference to the diversity of indigenous wild-growing populations that are the ancestors of the currently cultivated forms.

## 2. Results and Discussion

The results obtained confirm our previous observations concerning the variability in the functional traits of valerian. The weight of the roots collected from wild-growing populations was over three times lower than the mass of the raw materials collected from ‘Lubelski’ forms (22.66 and 74.37 g DW per plant, respectively). However, the variability (expressed as the coefficient of variation, CV) between wild-growing populations and between cultivated forms was similar (27 and 25%, respectively). The four forms of the ‘Lubelski’ cultivar differed markedly from each other in the root weight (from 48.7 to 90.74 g DW per plant), and they were also strongly internally differentiated concerning this trait (CV: from 49 to 75%) (Table 1). As a result, the diversity between the forms was lower than the diversity within the forms. The L2 form appeared to be the most uniform in terms of this trait, indicating the better efficiency of the selection work carried out by farmers in the region of its origin. Although cultivated on a large scale, the data concerning the yields of the ‘Lubelski’ cultivar are relatively scarce. According to Wiśniewski et al. (2016), the dry mass of the ‘Lubelski’ underground organs amounts to 63.9 g per plant, which is comparable with our results; however, no data on the diversity of this trait among ‘Lubelski’ plants are available [16].

The results obtained in our work also illustrate the effect of ploidy on the functional traits of valerian. The basic chromosome number of *V. officinalis* is x = 7 [17]. Among the species, diploids (2x = 14), tetraploids (4x = 28), and octoploids (8x = 56) have been repeatedly found [3,18,19,20]. Recently, a hexaploid population (6x = 42) and populations consisting of individuals with different numbers of chromosomes have been identified as well [3,21]. In our experiment, the plants originating from wild-growing populations were diploids, while all the ‘Lubelski’ forms were identified as pure tetraploids (Table 2). It can be assumed that the higher root weight of the cultivated tetraploid forms was related to the higher number of chromosomes, which was pointed out earlier by many authors [10].

According to the European Pharmacopoeia [5], the content of sesquiterpenic acids in the dried, uncut, or fragmented roots should not be lower than 0.17%. Among these acids, the most important ones are valerenic (VA) and acetoxyvalernic acid (AVA). Therefore, this group of compounds (sesquiterpenic acids) is commonly known as valerenic acids. The content of VA seems to be crucial here because, in clinical studies, the anxiolytic activity of the compound was confirmed [12]. In our study, the content of these compounds was distinctly lower in wild-growing populations (0.013%) compared to cultivated forms (0.321%). In contrast to the weight of the roots, the differences concerning this trait between wild-growing populations were several times larger (CV 59%) than the variation between cultivated forms (CV 18%). The content of valerenic acids varied between forms from 0.281 (L1) to 0.394% (L2). However, the forms were strongly internally differentiated in this trait (CV from 18 to 55%). Taking into consideration the above-mentioned anxiolytic activity of VA, the L2 form is highly notable, being characterized by high contents of both VA (0.209%) and AVA (0.188%) (Table 3).

Very low concentrations of these compounds in Polish wild-growing populations were presented in our previous paper [22]. This was also shown by Nakurte et al. (2020) in their research on populations originating from Latvia [23]. The content of valerenic acids in these populations varied from 0.002 to 0.014%. This low content was probably related to their ploidy level (diploids). This phenomenon was described earlier by Bernath (1970) [24]. According to his results, higher chromosome numbers in valerian result in higher contents of valerenic acids. While the contents of these substances were as low as 5 and 32 mg% for diploid and tetraploid plants, respectively, the contents in individual octoploid valerian varied between 138.9 and 264.7 mg% [24].

When considering the ‘Lubelski’ cultivar, the contents of valerenic acids in the investigated four forms were relatively high in comparison with the results of other authors. In the research of Nurzyńska-Wierdak (2014) on ‘Lubelski’ roots obtained from commercial cultivation, the valerenic acids content amounted to 0.12% [25]. According to Wiśniewski et al. (2016), the content of these compounds in ‘Lubelski’ cultivar varied from 0.11 to 0.25%, depending on the part of the underground organs analyzed (rhizome or roots) and the method of cultivation [16]. In the case of valerian, the term of establishing the plantation, harvest term, postharvest treatment, and conditions of storage may influence this trait as well [26,27,28,29].

Another important quality discriminant in valerian roots is the content of EOs. With reference to the European Pharmacopoeia, valerian’s content should not be lower than 4 mL × kg^−1^ DW in whole or fragmented drug forms. In our work, in contrast to the valerenic acids, the content of EOs in wild-growing populations was almost twice as high (7.62 mL × kg^−1^ DW) than in cultivated forms (4.35 mL × kg^−1^ DW). The diversity concerning this trait between wild-growing populations and between cultivated forms was relatively low (CV 25 and 12%, respectively). Among the four ‘Lubelski’ forms, the contents varied from 3.87 (L3) to 4.68 (L2) mL × kg^−1^ DW. Higher diversity was observed within individual forms (CV from 28 to 57%) (Table 4).

In general, the results obtained in our work correspond with the literature data [23,29,30]. Higher contents of EOs in wild-growing populations originating from Central Europe compared to valerian cultivars were confirmed earlier by Bączek et al. [22] and Nakurte et al. [23]. The populations from Serbia and Hungary were also a rich source of EOs (18.8 and 6.0 mL × kg^−1^ DW, respectively) [31,32].

Regarding valerian varieties or forms cultivated in Europe, previous research indicates great variability in the EO contents. For example, Letchamo et al. [30] provided the values of 8.7 mL × kg^−1^ DW for variety ‘Select’ and 11.0 mL × kg^−1^ DW for ‘Anthon’. Seidler-Łożykowska et al. [29] showed 11.5 mL × kg^−1^ DW in the case of ‘Polka’, while Nurzyńska-Wierdak [25] reported the value of 20.6 mL × kg^−1^ DW for ‘Lubelski’. In this context, the content of the EOs in the ‘Lubelski’ forms used in our experiment seems rather low. Moreover, the L3 form did not meet the Eur Pham. requirements concerning the level of EO content. The most promising form here seems to be the L2 form, which was characterized by a relatively high and stable content of EOs (4.68 mL × kg^−1^ DW).

In the present study, 44 compounds were identified in the EOs, comprising up to 98.7% of the total fraction (Table 5 and Table 6). Both oxygenated monoterpenes and sesquiterpenes were the main groups in the samples analyzed. Oxygenated monoterpenes were represented mainly by bornyl acetate (29.08% in wild-growing populations, 15.61% in the ‘Lubelski’ forms), while α-fenchene constituted a fundamental part of the monoterpene hydrocarbons (27.64 and 20.69%; respectively). Within oxygenated sesquiterpenes, valerenal was found in the highest amount (18.88% in wild-growing populations, 15.55% in the cultivated forms). Among the above-mentioned dominant compounds, bornyl acetate strongly diversified EOs originating both from wild-growing populations and cultivated forms (Table 5 and Table 6). According to Nakurte et al. [23], wild-growing populations were characterized by higher amounts of bornyl acetate in EOs compared to cultivated forms, followed by a slightly lower share of valerenal [23]. This relationship was also observed in our work.

Here, high variability concerning the EO compositions was observed both within individual wild-growing populations and within the cultivated forms, with CV reaching up to 200%. Such high diversity was described earlier by Raal et al. [33]. This phenomenon may be explained by the polymorphic character of valerian. In general, the chemical profiles of the analyzed EOs correspond well with the results of other authors. The domination of bornyl acetate in valerian EO, followed by valerenal, was reported previously by Raal et al. [33], Baranauskiene [34], and Nakurte et al. [23].

Among sesquiterpenes, three ring forms are unique for valerian species, namely: kessane, valerenal, and eleman types. It is worth noting that all these forms were detected in examined EOs and represented by the following compounds: kessane and its acetate, valerenal and its acetate, and valeranone. According to Houghton et al. [10], three chemotypes of valerian were distinguished based on the percentage share of specific sesquiterpenes, namely: kessane, valerenal, valeranone, and elemol. However, recent studies indicate that monoterpenes should also be taken into consideration when valerian chemotypes are recognized. Thus, the EOs analyzed in the present work can be classified as mixed bornyl acetate/valerenal chemotypes, which seem to be the most common in Europe, both for wild-growing and cultivated valerian. Interestingly, results provided by Safaralie et al. [35] show that the dominant compound of EOs from wild-growing Iranian populations is isovaleric acid (up to 41.8%). This compound, followed by its derivatives (e.g., valeric and 3-methylvaleric acids), was also detected in the present study, both as a free acid (up to 6.22%) and as an ester component. Some authors indicate clear domination of valerianol (>50%) in the valerian EOs [31]. However, this compound was not identified in our study.

## 3. Materials and Methods

### 3.1. Plant Materials

#### 3.1.1. Cultivated Forms of the ‘Lubelski’ Cultivar

In this study, the local cultivar ‘Lubelski’ was represented by four cultivated populations which were selected by farmers and cultivated in four regions of Poland. These populations were designated with the symbols L1, L2, L3, L4 and referred to in this paper as ‘Lubelski’ forms.

**Figure 1:**
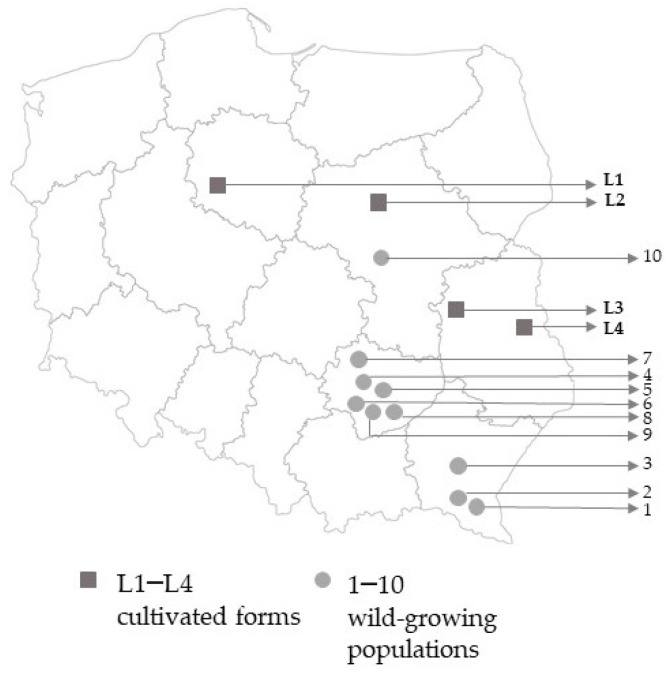
The origins of the wild-growing populations and the cultivated forms of ‘Lubelski’ cultivar.

The seeds of ‘Lubelski’ forms originated from the Kuyavian (L1), Masovian—northern part (L2), Mazovian—eastern part (L3), and Lublin (L4) voivodships in Poland (Figure 1). In each region, reproduction of the seeds was carried out based on the ‘Lubelski’ cultivar. The farmers declared that each year the selection treatments are carried out at their seed plantations mainly in terms of the size of underground organs and the general fitness of the plants. Therefore, it was assumed that locally cultivated populations of this cultivar differ significantly.

The seedlings of these ‘Lubelski’ forms were produced in the late summer and planted in September in the experimental field of the Warsaw University of Life Sciences WULS–SGGW, located in Wilanów (central Poland), in loamy soil. The plants were established with a spacing of 40 by 60 cm. In the spring of the next year, they were side dressed with fertilizer (N:P:K; 15:10:10; 70 kg ha^−1^) and harvested in the autumn, 16 months after seed germination. Hand weeding was performed during the whole cultivation process. The plants were harvested manually from the field, then the underground organs were cut in half to facilitate drying, then washed with high-pressure water. From each form, 30 randomly selected plants were collected and dried separately at 40 °C. The dry mass of the roots was recorded. The raw material from each plant was analyzed separately and the mean values per form were provided in Chapter 3. Soil and climatic parameters are presented in Table 7 and Table 8.

#### 3.1.2. Wild-Growing Populations

Wild-growing valerian populations used in the study originated from central and south-eastern Poland, namely: the Central Mazovian Lowlands, Kielce Upland, Nida Basin, and Bieszczady Mountains (Figure 1). Their seeds were collected from at least 30 individuals per population (forming one sample of the seeds per population). The populations (accessions) were introduced into the Collection of Medicinal and Aromatic Plants of the Polish GeneBank. For the study, ten populations were selected. Their origin is described in Table 9. The populations are available under accession numbers given in Table 9. From each population, 30 randomly selected seedlings were planted (the germinability of the seeds was at a level of 92–86%). The process of establishing the field experiment with these populations and the production of plant material were identical to those applied for ‘Lubelski’ forms (Section 3.1.1). In the autumn, ten randomly selected plants from each population were harvested manually, cut in half to facilitate drying, washed, and dried at 40 °C. The dry mass of the roots was determined. From each population, one sample, developed on the basis of ten plants, was prepared.

### 3.2. Ploidy Test

**Figure 2:**
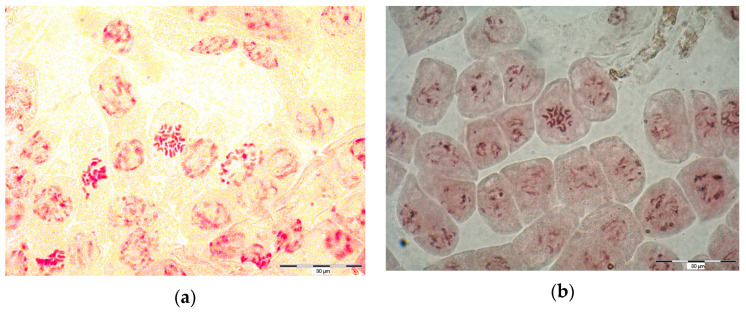
(**a**) Chromosomes of subpopulation L2; (**b**) Chromosomes of wild growing population no. 8.

Meristems of valerian root growth tips were collected from seeds germinating in Petri dishes on filter paper. In order to shorten the chromosomes, the plant material was treated with ice water for 2 h at 4 °C or with 0.002 M 8-hydroxyquinoline solution for 3 h at 16 °C. The roots were then fixed in a mixture of absolute ethyl alcohol and glacial acetic acid (3:1) for 24 h. After fixation, the material was transferred to 70% ethyl alcohol and stored in a refrigerator (4 °C) until observation. Before the preparation of microscopic slides, the samples were stained for an hour in a 1% solution of orcein in 45% acetic acid and hydrolyzed for several minutes in 1 N HCl solution at 40 °C. Then, under a stereoscopic microscope, the root growth tips were cut off (approx. 0.5 mm), transferred to glass slides, and placed in a drop of 45% acetic acid with the addition of glycerine (9:1). After being covered with a coverslip, the samples were gently crushed. Metaphase plates were analyzed under a microscope (Eduko) and the number of chromosomes was determined. Photographs were taken with a digital camera using an AX Provis microscope, Olympus (Figure 2).

### 3.3. Chemical Analysis

#### 3.3.1. Content of Valerenic Acids

The air-dry, powdered, and homogenized samples (1.000 g) were placed into a conical flask and extracted with 10 mL of methanol (Sigma-Aldrich, Poznan, Poland) for 10 min at 40 °C in an ultrasonic bath (Sonic 6, Polsonic, Warsaw, Poland). The extract was filtered into a volumetric flask with a cotton bud. The final obtained extract was filtered into amber glass vials with a PTFE 0.22 μm pore and 25 mm diameter syringe tip filter (Sigma-Aldrich, Poznan, Poland).

**Figure 3:**
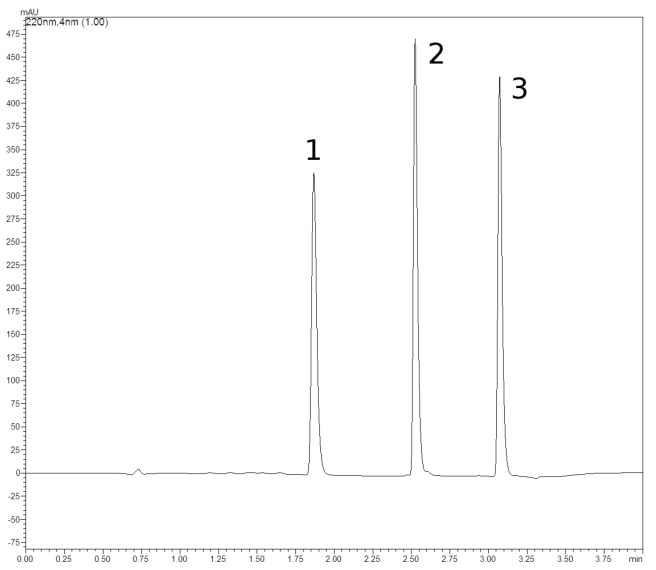
Chromatogram of valerenic acids standards. 1—hydroxyvalerenic acid; 2—acetoxyvalerenic acid; 3—valerenic acid.

**Figure 4:**
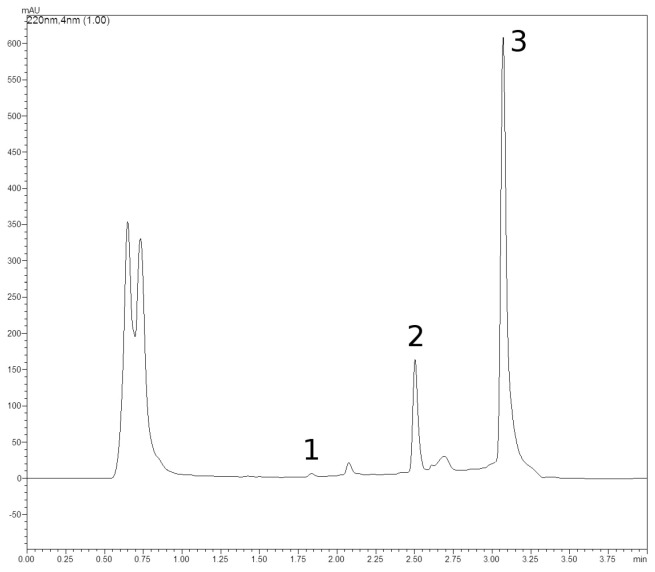
Chromatogram of valerian root extract. 1—hydroxyvalerenic acid; 2—acetoxyvalerenic acid; 3—valerenic acid.

Commercially available standards (ChromaDex^®^, Irvine, CA, USA) were separately dissolved with methanol (Sigma-Aldrich, Poznań, Poland) in 25 mL volumetric flasks according to the ChromaDex’s Tech Tip 0003: Reference Standard Recovery and Dilution; these were used as standard stock solutions [36]. Working solutions were prepared by diluting 10 µL and 100 µL of standard stock solutions with methanol in 10 mL volumetric flasks, 500 µL and 1000 µL in 5 mL volumetric flasks, and 1000 µL and 1000 µL of methanol. The working solutions and undiluted stock solutions were injected (1 μL) into a column in six replicates (n = 6) using SIL-20AC HT. Six-point calibration curves were plotted according to the external standard method by correlating the concentration with the peak area. Curve parameters were calculated with Microsoft Excel 14. The signal-to-noise ratio approach was used to determine LOD (S/N of 3:1) and LOQ (S/N of 10:1). The peak table and UV-spectra library (190–450 nm) of individual compounds were also created (Table 10, Figure 3 and Figure 4).

The quantitation was performed using a Shimadzu Prominence chromatograph equipped with an SIL-20A auto sampler, an SPD-M10A VP PDA photodiode array detector, and CLASS VP™ 7.3 chromatography software (Shimadzu, Kyoto, Japan). Separations were achieved using a C18 reversed-phase, 100 mm × 4.60 mm column with 2.7 μm core-shell particles (ReproShell ODS-3 by Dr Maisch, Ammerbuch, Germany). A binary gradient of deionized water adjusted to pH 3 with phosphoric acid (Sigma-Aldrich, Poznań, Poland) as phase A and ACN (Sigma-Aldrich, Poznań, Poland) as phase B was used as follows: 0.01–0.5 min—45% B; 2.0–2.1 min—90% B; 2.3–4.00 min—45% B; 4.0 min—stop, flow rate 1.5 mL × min^−1^, oven temperature 45 °C, injection volume 1 μL.

Compound identification was carried out through comparisons of retention time as well as UV-spectra with standards. The content of the determined compounds is expressed as a percentage. The results were analyzed with one-way ANOVA according to Tukey’s HSD test at an α = 0.05 significance level using Stat Graphics Plus for Windows v. 4.1 software.

#### 3.3.2. Content of EOs

The isolation of EOs was carried out using the hydrodistillation method in a Clevenger-type apparatus according to the European Pharmacopoeia *Valerianae radix* monograph 07/2015:0453. Air-dried powdered raw material weighing 40 g was used in a 2 L round-bottomed flask with 500 mL of water as the distillation liquid. The analysis was performed in triplicate. To solubilize the EO, 0.5 mL of xylene was added. The content of EOs was measured and expressed in mL × kg^−1^ DW.

#### 3.3.3. EO Compositions (GC-MS, GC-FID)

The qualitative GC-MS analysis of the EOs was performed on an Agilent GC-MS system consisting of a 7820A gas chromatograph equipped with an HP-20M capillary column (25 m × 0.32 mm i.d., film thickness 0.30 µm) and a 7693 autosampler connected to a mass spectrometer (Agilent 5977B, Santa Clara, CA, USA) controlled by MassHunter Acquisition software, Verion 10.0 Sr1 GC (Santa Clara, CA, USA). Helium was used as a carrier gas at a flow rate of 1.5 mL × min^−1^. The injector temperature was 250 °C and the MS source temperature was 230 °C. The oven temperature was programmed to rise from 60 (5 min) to 200 °C at a rate of 2.5 °C × min^−1^, then from 200 (10 min) to 220 °C at a rate of 15 °C × min^−1^, where it was held for 10 min. The injection volumes were 1.0 µL. The split injection was conducted with a split ratio of 1:30. The mass spectra were recorded at 70 eV (EI) and were scanned in the range 35–400 *m*/*z*. The components were identified by comparing their real retention indices relative to the n-alkanes (C7–C30) and the mass spectra with the NIST 20 Mass Spectral Library (Gaithersburg, MD, USA).

The quantitative GC-FID analysis was performed using a Hewlett Packard 6890 gas chromatograph equipped with a flame ionization detector (FID) and capillary polar column HP 20M (25 m × 0.32 mm × 0.3 µm film thickness). The analysis was carried out using the following temperature programme: oven temperature isotherm was used at 60 °C for 2 min, then it was programmed to increase from 60 °C to 220 °C at a rate of 4 °C per min and held isothermally at 220 °C for 5 min. The injector and detector temperatures were, respectively, 220 °C and 260 °C. The carrier gas (He) flow was 1.1 mL × min^−1^. The split ratio was 1:70. Manual injection of 0.5 µL essential oil was applied. Component identification was confirmed via comparison of their retention times with those of pure authentic samples and by means of their linear retention indices (RI) relative to the series of n-hydrocarbons (C7–C30) under the same operating conditions. The percentage composition of the EOs was computed from the GC peak areas using the normalization method without the use of correction factors. RI values were calculated in accordance to following formula: RI = 100n_0_ + 100((RT_x_ − RTn_0_)/(RTn_1_ − RTn_0_)), where RI is the retention index, RT is the retention time, *x* is the target compound, *n*_0_ is the n-alkane directly eluting before *x*, and *n*_1_ is the n-alkane directly eluting after *x*.

### 3.4. Statistical Analysis

The observed data were evaluated through factorial analysis of variance (ANOVA), and the mean differences were compared using post hoc test at a significance level of α = 0.05 according to Tukey’s HSD. The standard deviation (±SD) and coefficient of variation (CV%) were estimated. Statistical analyses were performed using STATISTICA 12.5 (TIBCO Software Inc., Palo Alto, CA, USA).

## 4. Conclusions

The obtained results indicate distinct differences between the wild-growing populations and cultivated forms of the ‘Lubelski’ cultivar with respect to the mass of underground organs and the content of valerenic acids and EOs. The wild-growing populations were characterized by distinctly lower masses of raw materials and lower contents of valerenic acids than the ‘Lubelski’ forms, whereas the contents of EOs were significantly higher in wild-growing populations. Taking into consideration the intraspecific diversity, much greater variability was observed within particular forms than between forms of the ‘Lubelski’ cultivar. This was visible both with respect to the mass of underground organs and the content of analyzed biologically active compounds. Among the studied forms of ‘Lubelski’ cultivar, the L2 form was found to be especially interesting, since it was characterized by a relatively high yield of the roots and high content of valerenic acids and EOs. What’s more, the diversity of this form was the lowest when compared to others. This means that the L2 form is the most interesting from the perspectives of further selection and agrotechnical works.

## Figures and Tables

**Table 1:** Dry mass of underground organs (g × plant^−1^).

Origin of Plants		SD	CV
Wild-growing	22.66	±6.19 b	27
L1	76.35	±50.14 ab	66
L2	81.68	±39.98 a	49
L3	90.74	±68.42 a	75
L4	48.70	±24.19 ab	50
Mean L1–L4	74.37	±18.54	25

L1–L4: forms of ‘Lubelski’ cultivar; SD—standard deviation; CV—coefficient of variation. Means followed by the same letters are not significantly different at *p* < 0.05 according to Tukey’s HSD.

**Table 2:** The number of chromosomes.

Origin of Plants	Ploidy Level
Wild-growing	2n = 2x = 14
L1–L4	2n = 4x = 28

L1–L4: forms of ‘Lubelski’ cultivar.

**Table 3:** The content of valerenic acids (%).

Origin of Plants	VA	SD	CV	AVA	SD	CV	VA + AVA	SD	CV
Wild-growing	0.011	±0.006 c	56	0.002	±0.001 b	89	0.013	±0.007	59
L1	0.135	±0.036 ab	27	0.146	±0.034 a	23	0.281	±0.052	18
L2	0.209	±0.113 a	54	0.188	±0.100 a	53	0.397	±0.184	46
L3	0.123	±0.081 b	66	0.173	±0.103 a	59	0.296	±0.163	55
L4	0.130	±0.084 b	64	0.179	±0.068 a	38	0.309	±0.111	36
Mean L1–L4	0.149	±0.032	21	0.171	±0.032	19	0.321	±0.059	18

L1–L4: forms of ‘Lubelski’ cultivar; VA—valerenic acid; AVA—acetoxyvalerenic acid; SD—standard deviation; CV—coefficient of variation. Means followed by the same letters in columns are not significantly different at *p* < 0.05 according to Tukey’s HSD.

**Table 4:** The content of essential oil (mL × kg^−1^ DW).

Origin of Plants		SD	CV
Wild-growing	7.62	±1.92 a	25
L1	4.49	±2.55 b	57
L2	4.68	±1.30 b	28
L3	3.87	±1.85 b	48
L4	4.36	±1.59 b	36
Mean L1–L4	4.35	±0.53	12

L1–L4: forms of ‘Lubelski’ cultivar; SD—standard deviation; CV—coefficient of variation. Means followed by the same letters are not significantly different at *p* < 0.05 according to Tukey’s HSD.

**Table 5:** Chemical composition of the EOs of wild-growing populations (% area).

	Compounds	RI	Wild-Growing Populations										Mean	SD	CV
			1	2	3	4	5	6	7	8	9	10			
**1**	α-pinene	1023	1.15	0.20	0.35	0.13	1.05	1.71	0.92	0.54	0.38	0.87	0.73	±0.50	68
**2**	α-fenchene	1055	15.32	12.33	18.96	15.05	18.90	27.64	22.03	17.17	18.52	21.24	18.72	±4.29	23
**3**	β-pinene	1067	0.28	0.58	0.44	0.09	0.21	0.17	0.17	0.29	1.05	0.36	0.36	±0.28	77
**4**	α-terpinene	1107	0.02	0.01	0.05	n.d.	0.02	0.01	0.02	0.05	0.01	0.02	0.02	±0.02	68
**5**	limonene	1118	0.09	0.10	0.13	0.45	0.09	0.23	0.16	0.17	0.22	0.04	0.17	±0.12	69
**6**	β-phellandrene	1157	0.07	0.04	0.05	0.10	0.03	0.02	0.01	0.02	0.01	0.05	0.04	±0.03	72
**7**	γ-terpinene	1173	0.02	0.03	0.01	0.02	0.02	0.02	0.01	0.03	0.01	0.01	0.02	±0.01	44
**8**	*p*-cymene	1195	0.02	0.04	0.01	0.05	0.03	0.02	n.d.	n.d	0.02	0.01	0.03	±0.01	57
**9**	n-hexyl isovalerate	1204	0.02	0.01	0.03	0.05	0.05	0.01	0.04	0.02	0.02	0.06	0.03	±0.02	58
**10**	δ-elemene	1234	0.12	0.02	0.15	0.16	0.38	0.02	0.54	0.27	0.09	0.41	0.22	±0.18	82
**11**	α-copaene	1257	0.01	0.02	0.01	0.01	0.02	0.02	0.01	0.03	0.01	0.02	0.02	±0.01	44
**12**	α-gurjenene	1269	0.07	0.02	0.01	0.03	n.d.	0.03	0.04	0.01	0.01	0.01	0.03	±0.02	79
**13**	bornyl acetate	1443	29.08	27.13	19.22	24.18	25.02	17.95	16.13	28.99	22.79	23.16	23.37	±4.49	19
**14**	thymol, methyl ether	1452	0.02	0.06	0.04	0.05	0.03	0.10	0.04	0.03	0.05	0.01	0.04	±0.02	58
**15**	(E)-β-caryophyllene	1464	0.87	0.72	0.83	0.64	1.01	0.69	0.73	0.64	0.35	0.42	0.69	±0.20	29
**16**	β-gurjunene	1485	n.d	n.d	n.d	0.03	0.64	n.d.	0.03	0.09	0.19	n.d.	0.20	±0.26	131
**17**	humulene	1499	2.19	3.33	0.97	3.18	0.65	0.79	4.62	3.16	4.87	5.23	2.90	±1.71	59
**18**	γ-muurolene	1507	n.d	n.d.	0.01	0.20	0.01	0.01	0.04	0.01	0.09	0.06	0.05	±0.07	123
**19**	myrtenyl acetate	1514	5.06	3.90	5.23	6.17	4.62	3.16	5.41	6.70	3.21	4.15	4.76	±1.18	25
**20**	isovaleric acid	1524	0.60	0.16	1.12	1.37	1.15	0.62	0.74	1.00	0.82	0.53	0.81	±0.36	44
**21**	α-terpineol acetate	1562	0.55	0.73	0.18	0.93	0.42	0.73	0.65	0.58	0.71	0.44	0.59	±0.21	35
**22**	valencene	1575	1.30	2.15	1.78	1.43	0.91	1.06	3.15	2.06	1.20	2.96	1.80	±0.78	43
**23**	borneol	1578	4.59	3.33	5.70	4.04	3.98	3.13	2.27	3.12	4.50	3.73	3.84	±0.96	25
**24**	kessane	1580	0.02	n.d	n.d	0.01	0.02	0.02	0.01	n.d.	n.d.	n.d.	0.02	±0.01	34
**25**	germacrene D	1588	0.36	1.16	1.06	1.08	0.69	0.71	2.36	2.14	1.60	2.32	1.35	±0.72	53
**26**	myrtenol	1603	0.56	0.40	0.86	1.24	1.35	0.98	0.55	1.74	0.71	0.39	0.88	±0.45	51
**27**	3-methylvaleric acid	1627	0.02	0.01	0.01	n.d.	n.d.	0.03	0.60	0.18	0.05	0.13	0.13	±0.20	156
**28**	2.5-dimethoxy-p-cymene	1656	0.01	0.01	0.02	0.02	0.01	n.d.	n.d.	0.01	0.02	0.01	0.01	±0.01	38
**29**	β-ionone	1660	0.03	0.01	0.01	0.01	0.02	0.03	0.01	0.01	0.02	0.01	0.02	±0.01	53
**30**	myrtenyl isovalerate	1664	0.92	0.16	0.22	0.53	0.12	0.23	0.47	0.71	0.21	0.36	0.39	±0.26	67
**31**	caryophyllene oxide	1666	0.88	0.96	0.72	0.55	1.02	0.78	0.55	0.64	0.89	0.24	0.72	±0.24	33
**32**	pacifgorgiol	1682	1.76	2.54	1.06	2.09	0.98	1.77	1.90	1.52	2.16	1.84	1.76	±0.48	27
**33**	maaliol	1690	n.d.	n.d.	n.d.	0.02	0.06	0.02	0.03	0.24	0.18	n.d.	0.09	±0.09	106
**34**	ledol	1702	0.22	0.35	0.56	0.17	0.11	0.34	0.15	0.16	0.45	0.19	0.27	±0.15	55
**35**	valeranone	1747	1.45	1.11	2.20	1.69	1.46	2.15	1.74	1.23	2.14	3.18	1.84	±0.61	33
**36**	spathulenol	1774	2.32	3.06	2.26	0.90	1.70	2.04	3.11	0.65	3.81	3.17	2.30	±1.02	44
**37**	γ-eudesmol	1850	2.23	1.56	2.30	2.15	1.56	2.12	2.35	2.60	1.66	2.03	2.06	±0.36	17
**38**	valerenal	1903	18.88	13.90	16.01	11.86	15.79	17.08	8.55	7.17	8.53	5.16	12.29	±4.72	38
**39**	isospathulenol	1943	2.55	4.30	4.15	3.44	2.08	1.58	1.47	1.88	3.01	2.76	2.72	±1.01	37
**40**	*trans*-valerenyl acetate	1985	0.13	1.22	0.58	1.78	1.18	0.76	0.44	1.02	2.35	1.34	1.08	±0.66	61
**41**	kessanyl acetate	2002	0.61	4.65	3.28	5.01	3.87	3.19	5.33	5.72	3.74	2.99	3.84	±1.48	39
**42**	*cis*-valerenyl acetate	2022	0.53	0.15	0.67	0.76	0.54	1.06	0.60	0.02	0.01	n.d.	0.48	±0.36	74
**43**	palmitic acid	2044	0.12	0.04	0.01	0.20	0.03	0.03	0.05	0.01	0.01	0.01	0.05	±0.06	123
**44**	valeric acid	2091	3.65	6.01	2.32	4.06	5.52	4.35	8.30	4.18	4.98	6.22	4.96	±1.65	33
	Monoterpene hydrocarbones		16.97	13.33	20.00	15.89	20.35	29.82	23.32	18.27	20.22	22.60	20.08		
	Oxygeneted monoterpenes		39.86	35.55	31.23	36.61	35.42	26.05	25.05	41.16	31.97	31.88	33.48		
	Sesquiterpene hydrocarbones		4.92	7.42	4.82	6.76	4.31	3.33	11.52	8.41	8.41	11.43	7.13		
	Oxygeneted sesquiterpenes		29.85	31.27	32.74	28.35	29.41	31.17	24.34	21.34	26.79	21.07	27.63		
	Others		7.10	8.94	4.79	8.32	7.86	7.04	12.10	7.63	8.27	9.16	8.12		
	Total identified		98.70	96.51	93.58	95.93	97.35	97.41	96.33	96.81	95.66	96.14			

**Table 6:** Chemical composition of the EOs of ‘Lubelski’ forms (% area).

	Compounds	RI	Forms of ‘Lubelski’ Cultivar						
			L1	L2	L3	L4	Mean	SD	CV
**1**	α-pinene	1023	0.59	1.29	0.82	1.26	0.99	±0.34	35
**2**	α-fenchene	1055	17.93	15.90	20.69	17.74	18.07	±1.98	11
**3**	β-pinene	1067	0.21	0.53	0.35	0.52	0.40	±0.15	38
**4**	α-terpinene	1107	0.18	0.18	0.11	0.21	0.17	±0.04	24
**5**	limonene	1118	0.15	0.17	0.06	0.09	0.12	±0.05	44
**6**	β-phellandrene	1157	0.11	0.06	0.03	0.02	0.06	±0.04	74
**7**	γ-terpinene	1173	0.03	0.05	0.02	0.05	0.04	±0.02	42
**8**	*p*-cymene	1195	0.02	0.11	0.01	0.02	0.04	±0.04	110
**9**	n-hexyl isovalerate	1204	0.04	0.11	0.05	0.00	0.05	±0.05	92
**10**	δ-elemene	1234	0.77	0.54	0.14	0.13	0.39	±0.31	80
**11**	α-copaene	1257	0.68	0.64	0.51	0.55	0.59	±0.08	13
**12**	α-gurjenene	1269	0.46	0.59	0.39	0.47	0.48	±0.08	17
**13**	bornyl acetate	1443	15.56	15.40	15.61	13.11	14.92	±1.21	8
**14**	thymol, methyl ether	1452	1.39	1.03	0.65	0.85	0.98	±0.32	32
**15**	(E)-β-caryophyllene	1464	1.39	0.33	0.40	0.85	0.74	±0.49	65
**16**	β-gurjunene	1485	1.36	0.35	1.42	0.71	0.96	±0.52	54
**17**	humulene	1499	2.09	4.09	2.01	2.35	2.63	±0.98	37
**18**	γ-muurolene	1507	0.49	0.83	1.34	1.42	1.02	±0.44	43
**19**	myrtenyl acetate	1514	1.91	2.37	2.19	2.67	2.28	±0.32	14
**20**	isovaleric acid	1524	0.31	1.55	1.67	0.53	1.01	±0.69	68
**21**	α-terpineol acetate	1562	1.27	1.13	1.59	2.92	1.73	±0.81	47
**22**	valencene	1575	1.24	1.02	1.04	0.98	1.07	±0.12	11
**23**	borneol	1578	3.22	4.18	1.98	1.19	2.64	±1.32	50
**24**	kessane	1580	0.72	0.63	0.45	0.37	0.54	±0.16	29
**25**	germacrene D	1588	2.32	1.13	2.24	1.11	1.70	±0.67	40
**26**	myrtenol	1603	0.87	0.94	0.83	0.79	0.86	±0.06	7
**27**	3-methylvaleric acid	1627	0.52	0.69	0.73	0.33	0.57	±0.18	32
**28**	2,5-dimethoxy-p-cymene	1656	0.56	0.52	0.33	0.99	0.60	±0.28	47
**29**	β-ionone	1660	0.63	0.73	0.49	0.61	0.62	±0.10	16
**30**	myrtenyl isovalerate	1664	0.95	0.80	1.04	0.83	0.91	±0.11	13
**31**	caryophyllene oxide	1666	0.33	0.39	0.26	0.48	0.36	±0.10	26
**32**	pacifgorgiol	1682	1.80	2.28	1.70	1.95	1.93	±0.25	13
**33**	maaliol	1690	0.15	0.41	1.70	0.69	0.74	±0.68	92
**34**	ledol	1702	0.39	0.44	0.20	0.47	0.37	±0.12	33
**35**	valeranone	1747	0.07	0.32	0.67	1.13	0.55	±0.46	84
**36**	spathulenol	1774	1.62	2.57	1.83	2.21	2.06	±0.42	21
**37**	γ-eudesmol	1850	0.13	0.04	0.40	1.38	0.49	±0.61	126
**38**	valerenal	1903	12.35	13.55	10.67	12.05	12.16	±1.18	10
**39**	isospathulenol	1943	3.90	4.00	2.59	3.72	3.55	±0.65	18
**40**	*trans*-valerenyl acetate	1985	2.92	2.10	0.60	2.51	2.03	±1.01	50
**41**	kessanyl acetate	2002	4.92	0.27	1.35	2.91	2.36	±2.02	86
**42**	4(15)-selinene-11,12-diol	2022	0.96	1.00	0.51	0.99	0.87	±0.24	27
**43**	*cis*-valerenyl acetate	2044	0.64	0.34	0.17	0.73	0.47	±0.26	55
**44**	palmitic acid	2091	1.33	0.69	1.37	0.17	0.89	±0.58	65
**45**	valeric acid	2092	3.97	6.15	3.39	2.33	3.96	±1.61	41
Monoterpene hydrocarbones			19.22	18.29	22.09	19.91	19.88		
Oxygeneted monoterpenes			24.22	25.05	22.85	21.53	23.41		
Sesquiterpene hydrocarbones			10.80	9.52	9.49	8.57	9.60		
Oxygeneted sesquiterpenes			28.77	25.79	21.38	29.26	26.30		
Others			9.48	12.79	10.28	7.13	9.92		
Total identified			92.49	91.44	86.09	86.40			

**Table 7:** Soil parameters (macro- and micronutrient concentrations (mg × L^−1^).

pH	NO_3_^–^	NH_4_^+^	P	K	Ca	Mg	Cl	Na	Cu	Fe	Mn	Zn
6.78	63	12	82	158	727	127	42	42	2.9	49.7	5.7	5.3

**Table 8:** The climatic parameters of the year 2022.

Months	Temperature (°C)	Rainfall (mm)	Air Humidity (%)	Sun Days
April	8	21.8	69	23
May	16	8.2	62	31
June	21	22.5	66	26
July	23	20.8	60	29
August	25	9.0	55	30
September	16	7.8	60	28
October	14	3.5	69	31

**Table 9:** The origin and geographical coordinates of the studied valerian wild-growing populations.

No.	Region	Access. No.	Latitude	Longitude	Altitude	Place of Collection
1	Bieszczady Mountains	403180	N 49 23 95	E 022 08 78	491	Edge of the tracks
2	Bieszczady Mountains	403182	N 49 38 92	E 021 59 68	491	Wasteland
3	Bieszczady Mountains	403185	N 49 41 71	E 022 12 33	278	Meadow on a pond
4	Nida Basin	403186	N 50 43 33	E 020 31 53	256	Edge of a birch forest
5	Nida Basin	403188	N 50 46 66	E 020 31 23	256	Meadow
6	Nida Basin	403189	N 50 30 50	E 020 43 47	212	Extended wasteland
7	Kielce Upland	403191	N 51 06 13	E 020 15 91	233	A strip of wasteland along the road
8	Kielce Upland	403193	N 50 35 16	E 020 33 40	203	Meadow
9	Kielce Upland	403194	N 50 38 40	E 020 35 78	266	Wasteland near a drainage ditch
10	Central Mazovian Lowlands	403195	N 52 23 70	E 021 01 75	90	Meadow

**Table 10:** Validation parameters of the HPLC-DAD analysis (n = 6).

Compound	Rt (min)	λ (nm)	Precision Intraday (CV, %)	Interday Precision (CV, %)	Calibration Equation	R^2^ (n = 6)	Linear Range (mg × mL^−1^)	LOD (µg × L^−1^)	LOQ (µg × L^−1^)	Recovery (%)
Valerenic acid	3.05	220	0.81	1.52	y = 2428.3 x − 4414.6	0.9999	1.08–1018.00	19.08	63.60	98.3
Acetoxyvalerenic acid	2.50	220	0.47	1.05	y = 1792.7 x − 3608.7	0.9999	1.21–1218.92	26.32	87.76	102.9

## Data Availability

The data presented in this study are available in article.
